# PTSD and DNA Methylation in Select Immune Function Gene Promoter Regions: A Repeated Measures Case-Control Study of U.S. Military Service Members

**DOI:** 10.3389/fpsyt.2013.00056

**Published:** 2013-06-24

**Authors:** Jennifer A. Rusiecki, Celia Byrne, Zygmunt Galdzicki, Vasantha Srikantan, Ligong Chen, Matthew Poulin, Liying Yan, Andrea Baccarelli

**Affiliations:** ^1^Department of Preventive Medicine, School of Medicine, Uniformed Services University, Bethesda, MD, USA; ^2^Department of Anatomy, Physiology and Genetics, School of Medicine, Uniformed Services University, Bethesda, MD, USA; ^3^EpigenDx Inc., Worcester, MA, USA; ^4^Exposure, Epidemiology and Risk Program, Harvard School of Public Health, Harvard University, Boston, MA, USA

**Keywords:** post-traumatic stress disorder, DNA methylation, cytokines, inflammation, promoter region, Operation Iraqi Freedom, Operation Enduring Freedom, epigenetics

## Abstract

**Background:** The underlying molecular mechanisms of PTSD are largely unknown. Distinct expression signatures for PTSD have been found, in particular for immune activation transcripts. DNA methylation may be significant in the pathophysiology of PTSD, since the process is intrinsically linked to gene expression. We evaluated temporal changes in DNA methylation in select promoter regions of immune system-related genes in U.S. military service members with a PTSD diagnosis, pre- and post-diagnosis, and in controls.

**Methods:** Cases (*n* = 75) had a post-deployment diagnosis of PTSD in their medical record. Controls (*n* = 75) were randomly selected service members with no PTSD diagnosis. DNA was extracted from pre- and post-deployment sera. DNA methylation (%5-mC) was quantified at specific CpG sites in promoter regions of insulin-like growth factor 2 (IGF2), long non-coding RNA transcript H19, interleukin-8 (IL8), IL16, and IL18 via pyrosequencing. We used multivariate analysis of variance and generalized linear models to calculate adjusted means (adjusted for age, gender, and race) to make temporal comparisons of %5-mC for cases (pre- to post-deployment) versus controls (pre- to post-deployment).

**Results:** There were significant differences in the change of %5-mC pre- to post-deployment between cases and controls for H19 (cases: +0.57%, controls: −1.97%; *p* = 0.04) and IL18 (cases: +1.39%, controls: −3.83%; *p* = 0.01). For H19 the difference was driven by a significant reduction in %5-mC among controls; for IL18 the difference was driven by both a reduction in %5-mC among controls and an increase in %5-mC among cases. Stratified analyses revealed more pronounced differences in the adjusted means of pre-post H19 and IL18 methylation differences for cases versus controls among older service members, males, service members of white race, and those with shorter deployments (6–12 months).

**Conclusion:** In the study of deployed personnel, those who did not develop PTSD had reduced %5-mC levels of H19 and IL18 after deployment, while those who did develop PTSD had increased levels of IL18. Additionally, pre-deployment the people who later became cases had lower levels of IL18 %5-mC compared with controls. These findings are preliminary and should be investigated in larger studies.

## Introduction

Estimates of post-deployment PTSD prevalence in U.S. military service members who served in Afghanistan and Iraq are 12 and 18%, respectively (Hoge et al., 2004); a recent study reported averages of 10–20% (Thomas et al., 2010). Despite these high rates among military populations, as well as high rates in civilian populations [i.e., U.S. prevalence rates of 7–8% (Kessler et al., 1994, 1997); lifetime prevalence of 9–14% (Kessler et al., 1995; Breslau, 2001)], the underlying molecular mechanisms of PTSD are largely unknown.

Given the sustained stress of the sympathetic nervous system and resulting hyper-arousal state in PTSD, it has been hypothesized that immune system functioning is affected. A series of recent studies has shown that PTSD is associated with alterations in immune function (Hoge et al., 2009) in particular increased levels of some canonical pro-inflammatory cytokines (Spivak et al., 1997; Maes et al., 1999; Sutherland et al., 2003; von Kanel et al., 2007) and decreased levels of anti-inflammatory cytokines (Kawamura et al., 2001; von Kanel et al., 2007; Smith et al., 2011). Profiling using cDNA microarrays of peripheral blood during the triggering and development of PTSD in trauma survivors at the emergency room and 4 months later has found differential gene expression signatures in promoter regions of genes which distinguished PTSD patients (Segman et al., 2005; Zieker et al., 2007). Distinct expression signatures were found in particular for transcripts involved in immune activation (Segman et al., 2005). Up-regulated genes included insulin-like growth factor 2 (IGF2) and pro-inflammatory/neuroprotective chemokine, IL8 (CXCL8); down-regulated genes included the pro-inflammatory pleiotropic cytokines, interleukin 16 (IL16), and interleukin 18 (IL18) (Segman et al., 2005; Zieker et al., 2007). Similar findings of differential expression of genes involved in immune cell function were found among cases of PTSD from the World Trade Center attack versus controls (Sarapas et al., 2011) and in a study of Bosnian war refugees with PTSD versus controls (Nowotny et al., 2010).

An epigenetic mechanism, DNA methylation may play a significant role in the pathophysiology of PTSD, since the process is intrinsically linked to gene regulation. Recently, epigenetic mechanisms, including regulation of chromatin structure and DNA methylation, have been found to be regulators of gene transcription in the CNS (Zovkic and Sweatt, 2013). Methylation changes may completely silence a gene or may decrease or increase gene expression. It is hypothesized that epigenetic molecular mechanisms, especially DNA methylation/demethylation, may influence long-term behavioral change through active regulation of gene transcription in the CNS. A recent review proposed that epigenetic molecular mechanisms underlie the formation and stabilization of context- and cue-triggered fear conditioning based in the hippocampus and amygdala, a conclusion reached in a wide variety of studies using laboratory animals (Zovkic and Sweatt, 2013). In humans, there is a small, but growing body of literature that supports a potential link between DNA methylation of immune function genes and PTSD. A recent study applying methylation microarrays to assay CpG sites in peripheral blood from PTSD cases and controls found differential methylation in genes related to immune system functions (Uddin et al., 2010). In particular, this study found that IL8, a gene that regulates innate and adaptive immune system processes, was unmethylated among PTSD cases (Uddin et al., 2010). Another recent study in an African American population found that psychosocial stress may alter global and gene-specific DNA methylation patterns potentially associated with peripheral immune dysregulation (Smith et al., 2011).

In the present study, we focused on a set of growth factors/transcripts and cytokines/chemokines previously implicated in PTSD: paternally imprinted IGF2, maternally imprinted H19 (a long, non-coding RNA transcript), IL8, IL16, and IL18 (Segman et al., 2005; Zieker et al., 2007; Nowotny et al., 2010; Uddin et al., 2010; Sarapas et al., 2011; Smith et al., 2011). We evaluated promoter region methylation levels as percentage of 5-methyl cytosine (%5-mC) in DNA from serum of PTSD cases and controls, who were U.S. military soldiers who deployed to Afghanistan [Operation Enduring Freedom (OEF)] or Iraq [Operation Iraqi Freedom (OIF)] between 2004 and 2006. Our study had the unique ability to investigate temporal changes in methylation patterns after deployment [a proxy for exposure to a potentially traumatic event (PTE)]. The pre- and post-deployment samples collected in our study enable such evaluations of temporal changes in methylation patters. Our objectives were to investigate DNA methylation patterns associated with PTSD.

Since human studies of brain tissue are highly invasive, identifying a low-invasive biomarker of epigenetic patterns of PTSD would be of great clinical value. Most of the studies to date which have measured expression signatures or methylation patterns in PTSD or other psychiatric disorders have been carried out using peripheral blood (Segman et al., 2005; Zieker et al., 2007; Nowotny et al., 2010; Uddin et al., 2010; Sarapas et al., 2011; Smith et al., 2011). To our knowledge, serum has not yet been evaluated for gene-specific methylation patterns potentially associated with PTSD. Serum and cerebrospinal fluid (CSF) have been found to have good correlation with respect to cytokine gene expression, and in this exploratory study we evaluated DNA methylation from serum as a biomarker, without drawing links to other types of tissues or directly extrapolating its significance. Serum DNA methylation patterns may provide a surrogate indicator of differential response to stress and vulnerability to PTSD. Unlike previous studies, we had unique access to biologic samples prior to onset of disease and prior to deployment (a proxy for exposure to a PTE). Serum samples from military service members have been stored at the Department of Defense Serum Repository (DoDSR) since the early 1990’s. We were able to access those stored sera and identified samples drawn prior to and post-deployment for all cases and controls.

## Materials and Methods

### Study population

The study population has been described previously (Rusiecki et al., 2012). The target population was male and female U.S. Army and Marines service members serving their first OEF/OIF deployment between January 01, 2004 and December 31, 2006. Deployment length was between 6 and 18 months. Via query of medical records using the International Classification of Diseases, Ninth Revision (ICD-9) codes 290–320, we determined an absence of any mental health diagnoses dating back to at least 2 years prior to first OEF/OIF deployment for all cases and controls. In an effort to exclude possible confounding by other psychiatric illnesses, for which differential gene expression or methylation has been reported (Mill and Petronis, 2007; Shimabukuro et al., 2007; Tamura et al., 2007; Kuratomi et al., 2008; Feng and Fan, 2009; Iwamoto and Kato, 2009; Gavin and Sharma, 2010) post-deployment exclusion criteria for both cases and controls was ever having a health encounter for schizophrenia (ICD-9 code 295), bi-polar disorders (ICD-9 code 296), and manic phase bi-polar disorder (also ICD-9 code 296).

The PTSD cases (*n* = 75) had existing pre- and post-deployment serum samples housed at the DoDSR, met all the criteria above, and had at least two outpatient records with a primary diagnosis of chronic PTSD, based on ICD-9 Code 309.81 in the first diagnostic position. The first outpatient diagnosis was between 4 and 12 months after return from first deployment. The second outpatient diagnosis was any time after that, but within 2 years of return from first deployment. Additional criteria for inclusion as a case to this study was having one serum sample (the pre-deployment sample) drawn within 12 months prior to first OEF/OIF deployment and one sample (the post-deployment sample) drawn within 6 months after return from first OEF/OIF deployment. Cases were randomly selected within each gender, such that 25 females and 50 males were included. We identified an appropriate active duty service member control group (*n* = 75), who were frequency matched from a stratified, random sample based on age (20–26, 27–35), gender, and race, but for whom there was never a diagnosis of PTSD (ICD-9 Code = 309.81) or Traumatic Brain Injury (TBI) (ICD-9 Codes = 800.0–801.9, 803.0–804.9, or 850.0–854.1). Though cases in this study are presumed to have experienced a PTE by virtue of their PTSD diagnosis, the criteria of which requires exposure to a PTE, we have no data on formal assessment of trauma/PTE for either cases or controls. As such, this study uses deployment as a proxy for exposure to a PTE for both cases and controls. Because of Department of Defense policy regarding the DoDSR samples, we were unable to make contact with the study subjects for whom we had serum and medical encounter data.

### Sample preparation and laboratory methods

#### DNA extraction

For each PTSD case and control, The Armed Forces Health Surveillance Center identified a pre-deployment and a post-deployment serum sample and authorized release of up to 0.5 ml of serum per sample. These samples had been maintained at −30 °C continuously at the DoDSR. DNA was extracted using ChargeSwitch ^®^  gDNA 0.2–1 ml serum kit from (Invitrogen Carlsbad, CA, USA) and quantified via Qubit ^®^  dsDNA HS Assay Kit using a Qubit fluorometer (Invitrogen, Carlsbad, CA, USA).

#### Quantification of DNA methylation

DNA methylation was quantified via bisulfite treatment, PCR, and pyrosequencing. DNA was bisulfite treated using the Zymo DNA Methylation Kit (Zymo research, Orange, CA, USA). All assays used in this study were validated by PCR bias testing and sensitivity testing. This was done to assess the lowest input gDNA needed for bisulfite modification and the lowest input bisulfite converted DNA for PCR for each assay. For H19, IL8, IL16, and IL18, a minimum of 40 ng of DNA was used for bisulfite conversion. For IGF2, a minimum of 10 ng DNA was used for bisulfite conversion. The total DNA available for each sample was used as input gDNA for bisulfite conversion. Carrier RNA from Qiagen was used during the bisulfite modification and purification to minimize the loss of DNA during the procedure. Bisulfite treated DNA was eluted in 20 μl volume and 1 μl of it was used for each PCR. The PCR (45 cycles) was performed with one of the PCR primers biotinylated to convert the PCR product to single-stranded DNA templates. The PCR products (each 10 μl) were sequenced by Pyrosequencing PSQ96 HS System (Qiagen Pyrosequencing) following the manufacturer’s instructions (Qiagen Pyrosequencing). Pyrosequencing is a real-time sequencing-based on mutation analysis or methylation analysis technology. The methylation status of each CpG locus was analyzed individually as a T/C SNP using QCpG software (Qiagen Pyrosequencing).

**Figure 1:**
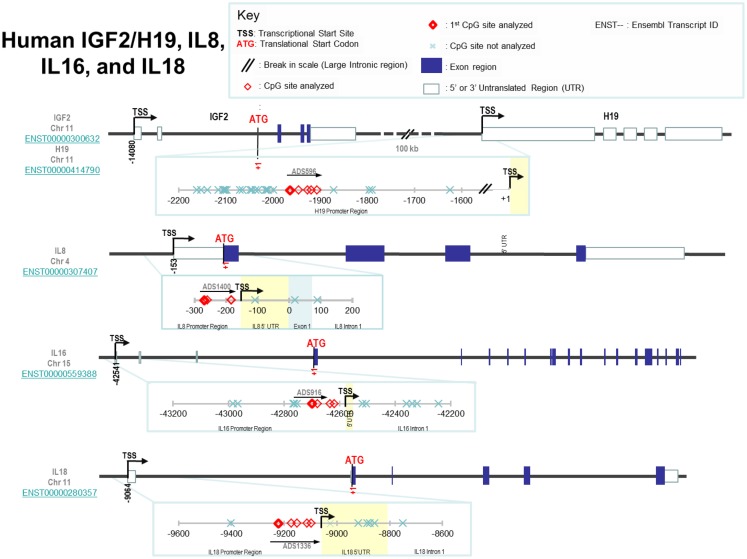
**Loci of specific CpGs measured in IGF2, H19, IL8, IL16, and IL18**.

The loci of specific CpGs measured in each promoter region are shown in Figure 1 and the rationale for selecting these specific loci was that they are generally located in the shore/vicinity of transcriptional start site. Loci measured were in the promoter regions of H19 [-1964, -1946, -1927, -1919 from the transcriptional H19 start site (TSS)], IGF2 [TSS-479, -476, -460, -361, -341, -322; note, the investigated regions for IGF2 covered the promoter of IGF2, p2, and the binding region for enhancer-blocking element CCCTC-binding factor (CTCF) upstream of the H19 start site], IL8 (TSS-116, -106, -31), IL16 (TSS-159, -139, -93, -79), and IL18 (TSS-158, -108, -86, -49, -33). The percentage of methylation (%5-mC) was expressed as 5-mC divided by the sum of methylated and unmethylated cytosine. This is interpreted as the percentage of cytosines at a given CpG site (or position) which is methylated. Percent 5-mC was measured at each CpG site, hereafter referred to as position, and a mean %5-mC was calculated across all positions measured in a promoter region. Starting from bisulfite modification, four controls [low, medium, high methylated DNA (EpigenDx, Inc.), and a no DNA template] were included in every pyrosequencing run to ensure specificity of PCR amplification, and success of pyrosequencing reactions.

DNA from cases and controls were randomly arranged on the plates for methylation quantification analysis. For 10% of the samples we included duplicates to which laboratory personnel were blinded, for the purpose of quality control (QC). Coefficients of variation as well as intraclass correlation coefficients (ICCs) were calculated for the means of each position in H19 (CV = 0.05, ICC = 0.75; *p* < 0.01), IL8 (CV = 0.01, ICC = 0.72; *p* < 0.01), IL16 (CV = 0.05, ICC = 0.68; *p* < 0.01), and IL18 (CV = 0.02, ICC = 0.89; *p* < 0.01). The duplicate samples were run on a separate plate from their counterparts, and some of the variation could be due to a plate effect.

Approximately 30% of the samples (*N* = 86) had DNA yields (e.g.,<40 ng) insufficient for bisulfite conversion of H19, IL8, IL16, and IL18. Approximately 5% of the samples (*N* = 15) had DNA yields (e.g.,<10 ng) insufficient for bisulfite conversion of IGF2. These samples were excluded from our study. For a small percentage of the samples for which bisulfite conversion was complete, pyrosequencing signals were too low to be reliable (0% for H19, 9% for IGF2, 0.05% for IL8, 8% for IL16, and 2% for IL18), thus we did not include them in our final analyses. It is unclear why signals were low for those particular samples, however, low signals were not associated with DNA yield. Distribution of samples either not measured or measured but with low signals did not differ by pre- and post-deployment or case/control status, with the exception of IGF2 pre-deployment case/control status. However, this is likely because there were only 16 with low signals for that assay, thus lending to the instability of those proportions.

#### Statistical methods

Our statistical analyses considered %5-mC in the promoter and/or known methylation region of each gene at each position (CpG site) measured as well as the mean of all the positions measured. We compared the change in cases’ %5-mC from pre- to post-deployment, to the change in controls’ %5-mC from pre- to post-deployment using multivariate ANOVA. We present the adjusted mean of pre-post methylation difference for cases versus controls, adjusted for age, gender, and race. To further investigate the adjusted means and differences between cases and controls, pre-deployment and post-deployment, we ran generalized linear models (GLMs) Using GLMs, we carried out case-case and control-control (i.e., pre- to post-deployment) comparisons, as well as case-control comparisons, pre-deployment, and post-deployment. We carried out the same analyses, stratifying by age, gender, race, and length of deployment, separately, to investigate any potential patterns within specific groups. SAS Version 9.2 was used to carry out all statistical analyses. The SAS procedures GLM and ANOVA were used to run our final models (SAS Institute Inc., 2008).

This study was approved by the Institutional Review Board at the Uniformed Services University of the Health Sciences.

## Results

Baseline characteristics of the study population are in Table 1. Although the study was designed with 75 cases and 75 controls, we excluded 2 study subjects (1 case/1 control) because their DNA yield was insufficient for DNA methylation quantification for any of the loci measured in both pre- and post-deployment time frames. The remaining 148 study subjects did not necessarily have complete data for all loci measured in both pre- and post-deployment time frames, but they did have DNA methylation quantified for at least one locus in at least one time frame. The study population did not differ by case-control status for age, gender, and race because of the selection and frequency matching criteria. Approximately 73% of cases and 76% of controls had deployments of 6 months to less than 12 months, while 27% of cases and 24% of controls had deployments of 12–18 months. These small differences were not statistically significant. The number of days between end of deployment and post-deployment serum draw ranged from 1 to 170 (mean = 22.45; SD = 38.84; median = 7), and the number of days between pre-deployment serum draw and start of deployment ranged from 58 to 358 (mean = 87.46; SD = 84.49; median = 58.5). There were no differences between cases and controls for these time intervals, nor were there differences by occupational code or rank/rate. There were four cases who also had a post-deployment diagnosis of TBI (defined as an ICD-9 code of 800.0–801.9, 803.0–804.9, or 850.0–854.1) (data not shown). We carried out all analyses including and excluding those four cases to evaluate potential confounding by TBI and found that results were very similar. We, therefore, present all results for analyses including those four PTSD cases with concurrent TBI.

**Table 1:** **Baseline characteristics of population**.

Characteristic	Cases (*N* = 74)		Controls (*N* = 74)		*p*-Value
	*N*	%	*N*	%	
**AGE**					
Younger (20–23 years)	40	54.1	38	51.4	0.74
Older (24–35 years)	34	46.0	36	48.7	
**GENDER**					
Male	49	66.2	49	66.2	1.00
Female	25	33.8	25	33.8	
**RACE**					
White	59	79.7	60	81.1	0.84
Black	15	20.3	14	18.9	
**DEPLOY LENGTH**					
Short (6 to<12 months)	54	73.0	56	75.7	0.71
Long (12–18 months)	20	27.0	18	24.3	
**TIME BETWEEN DEPLOYMENT END AND POST-DEPLOYMENT SERUM SAMPLE DRAW**					
≤7 days	41	55.4	38	51.4	0.62
>7 days	33	44.6	48	48.6	
**TIME BETWEEN PRE-DEPLOYMENT SERUM DRAW AND DEPLOYMENT START**					
≤90 days	52	70.3	45	60.8	0.23
>90 days	22	29.7	29	39.2	
OCCUPATIONAL CODING*					
Less likely involved in combat	19	38.8	20	38.5	0.97
More likely involved in combat	30	61.2	32	61.5	
Missing	25		22		
**RANK/RATE^#^**					
Junior enlisted	57	77.0	52	70.3	0.35
Middle-grade/senior enlisted and officer	17	23.0	22	29.7	

*Based on military occupational specialty POC coding as either less likely to be involved in combat or more likely to be involved in combat.

*^#^Junior enlisted included E-2 through E-4; middle/senior enlisted and officer included E-5 through E-8, O-1 through O-3, and W-2*.

Table 2 presents the results of a multivariate ANOVA comparing adjusted means of pre-post methylation differences for cases versus controls. These results show that, accounting for the exposure of deployment (i.e., proxy for PTE), there were significant reductions in %5-mC for controls, compared with cases at H19 (mean of positions 1–4: cases: +0.57%, controls: −1.97%; *p* = 0.04) and IL18 (mean of positions 1–5: cases: +1.39%, controls −3.83%; *p* = 0.01). Position 3 in H19 and positions 4 and 5 in IL18 had statistically significant differences between cases and controls, and though differences in the change of methylation were not significant at other positions for H19 and IL18, they all showed similar patterns.

**Table 2:** Multivariate ANOVA comparing adjusted* mean of pre-post methylation difference between cases and controls.

Gene promoter		*N*_pairs_^1^	Mean^2^	SE	*p*-Value
H19 (mean positions 1–4)	Case	37	0.57	1.10	0.04
	Control	45	−1.97	0.99	
Position 1; TSS-1964	Case	37	0.58	1.30	0.09
	Control	45	−1.92	1.16	
Position 2; TSS-1946	Case	37	1.38	1.41	0.14
	Control	45	−1.01	1.26	
Position 3; TSS-1926	Case	37	0.65	1.15	0.01
	Control	45	−2.93	1.03	
Position 4; TSS-1919	Case	37	−0.33	1.44	0.31
	Control	45	−2.01	1.29	
IGF2 (mean positions 1–6)	Case	52	−2.61	3.25	0.81
	Control	62	−3.49	3.01	
Position 1; TSS-479	Case	52	−0.22	4.77	0.76
	Control	62	−1.83	4.42	
Position 2; TSS-476	Case	52	−2.97	4.94	0.59
	Control	62	0.00	4.58	
Position 3; TSS-460	Case	52	4.16	4.93	0.40
	Control	62	−0.52	4.57	
Position 4; TSS-361	Case	52	−12.14	4.59	0.67
	Control	62	−9.98	4.32	
Position 5; TSS-341	Case	52	−2.79	3.97	0.84
	Control	62	−3.71	3.74	
Position 6; TSS-322	Case	52	−2.90	3.37	0.95
	Control	62	−3.13	3.17	
IL8 (mean of positions 1–3)	Case	36	−0.17	0.32	0.25
	Control	44	−0.58	0.28	
Position 1; TSS-116	Case	36	−0.54	0.54	0.38
	Control	44	−1.06	0.47	
Position 2; TSS-106	Case	36	−0.12	0.46	0.36
	Control	44	−0.58	0.40	
Position 3; TSS-31	Case	36	0.15	0.17	0.20
	Control	44	−0.10	0.15	
IL16 (mean of positions 1–4)	Case	31	−4.23	3.66	0.88
	Control	38	−3.61	3.66	
Position 1; TSS-159	Case	31	−5.61	4.80	0.97
	Control	38	−5.44	4.78	
Position 2; TSS-139	Case	31	−2.94	3.34	0.89
	Control	38	−3.44	3.33	
Position 3; TSS-93	Case	31	−4.29	6.58	0.95
	Control	38	−4.76	6.64	
Position 4; TSS-79	Case	31	−5.00	6.22	0.85
	Control	38	−3.78	6.27	
IL18 (mean of positions 1–5)	Case	37	1.39	1.85	0.01
	Control	45	−3.83	1.66	
Position 1; TSS-158	Case	37	−2.60	2.66	0.50
	Control	45	−4.63	2.40	
Position 2; TSS-108	Case	37	2.31	2.31	0.03
	Control	45	−3.50	2.08	
Position 3; TSS-86	Case	37	1.88	2.47	0.21
	Control	45	−1.66	2.22	
Position 4; TSS-49	Case	37	2.35	1.93	0.01
	Control	45	−3.43	1.75	
Position 5; TSS-33	Case	37	2.13	2.23	0.02
	Control	45	−4.03	2.02	

*Adjusted for age, gender, race.

*^1^Pairs of samples, i.e., pre and post paired samples among the cases and among the controls – number of pairs differ for the different promoter regions measured because of sensitivity of each assay – i.e., some samples were dropped because of low pyrosequencing signals for a specific assay*.

*^2^Adjusted mean of the difference from pre- to post-deployment for cases and for controls*.

Table S1 in Supplementary Material provides additional detail on the change in methylation across deployment for cases and controls; paired differences in adjusted mean levels for cases pre- and post-deployment and controls pre- and post-deployment, determined via GLM, are presented. The differences between cases and controls across deployment for H19 were driven primarily by the significant reduction in %5-mC among controls from pre- to post- deployment (the levels for cases were unchanged across deployment for H19). The pattern of decreased H19 methylation among controls from pre- to post-deployment is evident; statistically significant decreases were found at positions 1, 3, and 4, and for the mean of positions 1–4. The differences for IL18 were driven by both the decrease in methylation across deployment for controls as well as the increase in methylation across deployment for cases. There was a pattern of decreased methylation among controls from pre- to post-deployment, though a statistically significant decrease was found for position 5 only. There was also a pattern of increased IL18 methylation for cases pre- to post-deployment, though none of the comparisons was statistically significant.

Table S2 in Supplementary Material, which presents case-control comparisons for both pre- and post-deployment, further illustrates that for IL18, %5-mC was lower in cases compared with controls, pre-deployment (*p* ≤ 0.05 for positions 2, 4, and 5, and the mean of the five positions), while the pattern reversed post-deployment, such that levels were non-statistically significantly higher in cases compared with controls. Note that the term “case” in the pre-deployment context refers to people who later became cases, post-deployment; during the pre-deployment time period, they were not yet cases.

Also shown in Table S2 in Supplementary Material are a few other loci where significant differences in %5-mC were found between cases and controls pre-deployment, i.e., IGF2 position 2 (%5-mC_cases_ > %5-mC _controls_, *p* = 0.01) and IL8 position 2 (%5-mC_cases_ < %5-mC _controls_, *p* = 0.01). There were no significant differences in adjusted mean levels for the post-deployment case-control comparisons.

Stratified analyses (Table 3) revealed more pronounced differences in the adjusted means of pre-post H19 and IL18 methylation differences for cases versus controls among older (24–35 years) service members (H19 position 3 case/control: +0.13/−3.66; *p* = 0.05), males (H19 position 3 case/control: +0.80/−2.99; *p* = 0.01; IL18 mean case/control: +0.47/−5.38), service members of white race (H19 mean case/control: +0.72/−2.70; *p* = 0.01; IL18 mean case/control: +2.82/−2.16; *p* = 0.04), and those with deployments between 6 and 12 months (H19 position 3 case/control: +0.16/−3.20; *p* = 0.02; IL18 mean case/control: +2.52/−4.65; *p* = 0.01).

**Table 3:** Multivariate ANOVA comparing adjusted* mean of pre-post methylation difference for cases versus controls, stratified by age, gender, race, and deployment length.

Gene promoter/CpG site	Subject	Age: younger (20–23) and older (24–35)					
		Younger			Older		
		*N*	μ	*p*-Value	*N*	μ	*p*-Value
H19 position 3	Case	20	0.71	0.11	17	0.13	0.05
	Cont	25	−2.47		20	−3.66	
H19 mean of positions	Case	20	0.34	0.36	17	0.58	0.07
	Cont	25	−1.34		20	−3.00	
IL18 mean of positions	Case	20	0.60	0.22	17	1.38	0.05
	Cont	25	−3.94		20	−3.07	
		
		**Gender: female and male**					
		**Female**			**Male**		
		***N***	**μ**	***p*-Value**	***N***	**μ**	***p*-Value**
		
H19 position 3	Case	9	0.59	0.24	28	0.80	0.01
	Cont	16	−2.74		29	−2.99	
H19 mean of positions	Case	9	0.98	0.12	28	0.91	0.14
	Cont	16	−2.84		29	−1.27	
IL18 mean of positions	Case	9	1.12	0.35	28	0.47	0.03
	Cont	16	−1.80		29	−5.38	
	
		**Race: black and white**					
		**Black**			**White**		
		***N***	**μ**	***p*-Value**	***N***	**μ**	***p*-Value**
		
H19 position 3	Case	6	−0.65	0.43	31	1.64	0.01
	Cont	8	−3.07		37	−2.30	
H19 mean of positions	Case	6	−0.95	0.73	31	0.72	0.01
	Cont	8	0.14		37	−2.70	
IL18 mean of positions	Case	6	−0.31	0.21	31	2.82	0.04
	Cont	8	−5.53		37	−2.16	
	
		**Deployment length: long (6 to<12) and short (12–18)**					
		**Long Depl**.			**Short Depl**.		
		***N***	**μ**	***p*-Value**	***N***	**μ**	***p*-Value**
		
H19 position 3	Case	8	4.20	0.42	29	0.16	0.02
	Cont	11	0.75		34	−3.20	
H19 mean of positions	Case	8	3.56	0.38	29	0.12	0.12
	Cont	11	0.68		34	−2.14	
IL18 mean of positions	Case	8	−2.08	0.93	29	2.52	0.01
	Cont	11	−2.46		34	−4.65	

*For analyses stratified on age, adjusted for gender and race; for analyses stratified on gender, adjusted for age and race; for analyses stratified on race, adjusted for age and gender; for analyses stratified on deployment length, adjusted for age, gender, and race.

## Discussion

Our study found that there were significant differences in the change of methylation (%5-mC) across deployment between cases and controls for H19 and IL18. While there was no change in %5-mC in H19 for cases from pre- to post-deployment, there was a significant decrease among controls driving the difference in %5-mC change between the cases and controls. For IL18 there was a significant difference between the %5-mC decrease in controls and the %5-mC increase in cases, from pre- to post-deployment. These findings were more pronounced in both H19 and IL18 in younger service members, males, service members of white race, and those with shorter deployment length. There were a few additional statistically significant findings at specific CpG sites, such as pre-deployment case-control differences in IGF2 position 2 and in IL8 position 2. Given the low signals from the IGF2 assay being differentially distributed between pre-deployment cases and controls, we are cautious to interpret the results for IGF2. Whether these patterns represent markers of vulnerability or resiliency is speculative. Psychological stress incurred during deployment may be associated with a methylation response in these genes, but given the lack of PTE data for controls, this study is not able to address whether the response is protective of PTSD or puts one at risk for PTSD.

The most consistent result from our study, which is detailed in Table S2 in Supplementary Material, is that cases pre-deployment had significantly lower %5-mC in the IL18 promoter region than controls pre-deployment. This difference was found at positions 2, 4, and 5, and the mean of all five positions; *p*-values approached significance for positions 1 (0.08) and 3 (0.06). There was a reversal of this pattern post-deployment, in that cases had higher levels at all positions, compared to controls, though differences were not statistically significant. Given that DNA methylation is often inversely correlated with gene expression, in particular for immune system-related genes (Oliveira et al., 2009) these results for IL18 are generally consistent with findings from a previous study based on cDNA microarray investigation, which reported down-regulation of IL18 in PTSD cases (Segman et al., 2005). Interestingly, pro-inflammatory cytokine IL18, a key mediator of inflammation and immune response inducing interferon-γ (IFN-γ), is expressed in the brain and plays a significant role in a number of neuropathological disorders (Bossu et al., 2010; Anderson and Rodriguez, 2011). IL18 has been recently shown to attenuate breaks in the blood-brain barrier via IFN-γ independent pathway, suggesting its potential neuroprotective role (Jung et al., 2012). Our results of increased IL18 methylation in cases would imply down-regulation of the gene, which is consistent with previous findings (Zieker et al., 2007).

Our findings also suggest a potential role of H19 in stress response (23). The imprinted IGF2-H19 locus (CTCF binding cite) encodes the growth promoting hormone IGF2 and a long non-coding RNA H19. The maternally inherited locus transcribes untranslated H19 RNA, which plays the role of a trans-regulator of the imprinted gene network controlling embryonic growth and was recently shown to be processed into micro-RNA (miRNA)-675 (Cunningham et al., 2010; Keniry et al., 2012). Expression of both genes is partially regulated by a 3′ distal enhancer, and methylation of the CTCF binding site on the paternal chromosome prevents binding of the CTCF insulator allowing for activation of the IGF2 promoter. Lack of methylation of the CTCF binding site on the maternal chromosome, however, prevents IGF2 promoter activation (Fu et al., 2004; Cunningham et al., 2010; Keniry et al., 2012). Although a previous study reported that IGF2 was up-regulated in the whole blood (Zieker et al., 2007) IGF2-mediated signaling has been implicated in fear extinction and was proposed as a therapeutic venue to attenuate excessive fear memory potentially via promotion of neuronal survival and/or self-renewal of neural stem cells through its interaction with the insulin receptor (Agis-Balboa et al., 2011; Ziegler et al., 2012). This could suggest that it is not only IGF2 that may play a direct role in PTSD fear extinction, but H19 may be involved in the regulation of IGF2. Also, H19 or its miRNA products and inhibition of their putative targets may contribute to the stress response (23).

Previously, we investigated DNA methylation in repetitive elements, LINE-1 and Alu, in this population and found differential methylation between pre- and post-deployment controls and in cases versus controls, post-deployment. In the current study, we selected specific cytokine promoter regions of interest based on previous findings in the literature of differential gene expression or methylation of those cytokines between PTSD cases and controls (Segman et al., 2005; Zieker et al., 2007; Nowotny et al., 2010; Uddin et al., 2010; Sarapas et al., 2011; Smith et al., 2011). Increasing evidence for contribution by the chemokine network (Semple et al., 2010) and the known role of IGF2 (Torres-Aleman, 2010) in brain and vasculature development indicates it as an important target due to possible pre-morbid conditions. The neuro-chemokine IL8 has been implicated in brain development, neuroinflammation, and synaptic transmission (Rostene et al., 2011). Cytokine IL16, a chemo-attractant for immune cells expressing surface CD4 molecules (e.g., T-cells) (Cruikshank and Little, 2008) targets a number of neurophysiological membrane proteins, such as immediate-early gene c-Fos, potassium and calcium channels, *N*-Methyl-d-aspartate (NMDA)-receptor subunits, and neuronal phosphatases through its PDZ domain and exerts its autocrine function in the brain with a distinct role in neuropoiesis (Kurschner and Yuzaki, 1999; Schwab et al., 2001; Bannert et al., 2003). Chemokine IL8 and pro-inflammatory cytokine IL18 are known to induce inflammatory mediator interferon γ and have been found to play neuroprotective roles in models of neurodegenerative conditions (Ryu et al., 2010; Semple et al., 2010).

Only a handful of recent studies have evaluated gene-specific DNA methylation and PTSD (Uddin et al., 2010, 2011; Ressler et al., 2011; Trollope et al., 2012) to our knowledge, and these studies have used only post-PTE blood samples. A cross sectional study of PTSD-affected and -unaffected individuals enrolled in a longitudinal study which investigated methylation and immune function profiles in DNA derived from whole blood in a methylation microarray reported that immune system functions were significantly overrepresented among the genes uniquely unmethylated in those with PTSD (Uddin et al., 2010). This signature included IL8, a gene which regulates innate and adaptive immune system processes (Uddin et al., 2010). There is emerging evidence that chemokines like IL8 and its receptors not only regulate immune cell infiltration but also contribute to a variety of physiological functions underlying neurotransmission, neuro-protection, and neurogenesis (Rostene et al., 2011) and therefore may exert a neuro-modulatory role by changing the balance of neurotransmitters and affecting fear memory (Herry et al., 2008; Joels et al., 2008). However, a peripheral blood mononuclear cell (PBMC) gene expression profile carried out in trauma survivors found that IL8 was under-expressed in PTSD cases, which would indicate the potential for hypermethylation of IL8 in these cases (Segman et al., 2005). Another recent human study found that persons who experienced traumatic events were at increased risk for PTSD, but only those with lower methylation levels of a serotonin transporter gene, SLC6A4. At higher methylation levels, individuals with more traumatic events were protected from PTSD (Koenen et al., 2011). Our study did not find a post-deployment case-control difference in this gene. We did find a pre-deployment case-control difference in one position in IL8 (position 2) in that people who later became cases (pre-deployment cases) had lower levels of IL8 than did controls. This could indicate susceptibility to a PTE or to the development of PTSD, however we found it in only one position in the promoter region.

Serum has not previously been evaluated as a biomarker for DNA methylation patterns in cytokine gene promoter regions associated with PTSD. Most of the human studies which have investigated DNA methylation in PTSD have utilized predominantly whole blood-derived DNA (Uddin et al., 2010, 2011; Koenen et al., 2011; Ressler et al., 2011; Trollope et al., 2012). How correlated DNA methylation levels are in serum and whole blood to brain and other CNS tissues is not clear. However, it has been reported that some cytokines, such as IL18 can cross the blood-brain barrier (Alboni et al., 2010; Jung et al., 2012). The origin of circulating nucleic acids such as cell-free circulating DNA (cfcDNA) has been hypothesized to stem from necrosis or apoptosis (Gormally et al., 2007) though others report the possibility of an active release from cells (Anker et al., 1999; Stroun et al., 2001; Goebel et al., 2005; Rhodes et al., 2006). Excessive stress may induce DNA damage in the form of oxidized nucleosides, strand breaks, apoptosis, and necrosis and may be a source of cfcDNA in serum (Atamaniuk et al., 2011). Additionally, in the CNS, severe life stress leads to oxidative stress, and the accumulation of reactive oxygen species (ROS) is known to increase the susceptibility of brain tissue to damage (Schiavone et al., 2013). ROS, in turn, may also trigger molecular cascades leading to the blood-brain barrier permeability and neuronal death (Gu et al., 2011). Compared with cultured cells, clinical specimens, such as whole blood, serum, and even brain tissue and other CNS tissues, contain a heterogeneous mixture of cell types, each contributing its own unique methylation profile to the final analysis. Indeed DNA methylation patterns have been found to differ globally or locally, depending on the brain region/sub-region of focus (Iwamoto and Kato, 2009). One study demonstrated that the human cerebral cortex has an epigenetic signature distinct from the cerebellum (Ladd-Acosta et al., 2007) while others have shown that global DNA methylation differs across sub-regions of the rat hippocampus (Miller and Sweatt, 2007; Brown et al., 2008). These results taken collectively indicate that even different cortical cells are likely to have distinct epigenomic patterns. Serum DNA methylation patterns may provide a surrogate indicator of differential response to stress and PTSD, and our intent was to evaluate it as a non-invasive biomarker without drawing links to other types of tissues or directly extrapolating its significance. We evaluated difference in storage time of serum as a potential factor which may have influenced DNA methylation patterns. All samples for this study were obtained from the DoD Serum Repository in June 2009, and DNA was extracted from them soon thereafter. The time frame of serum being collected from our study subjects, based on the study sample criteria, was approximately January 2003 through June 2007. We found that cases and controls did not differ, either pre-deployment or post-deployment with respect to storage time. The mean storage time for cases pre-deployment was 4.5 years, for controls it was 4.8 years (*p* = 0.15). The mean storage time for cases post-deployment was 3.3 years, for controls it was 3.7 years (*p* = 0.14). As we expected, the storage time did differ by pre- and post-deployment time period. Thus for cases pre-deployment the mean was 4.5 years, while post-deployment it was 3.4 years (*p* = < 0.01). Likewise, the mean storage time for controls pre-deployment was 4.9 years, while post-deployment it was 3.7 (*p* < 0.01). Based on these differences, we adjusted all our models in which we carried out a pre/post comparison (i.e., Table 2; Table S1 in Supplementary Material) by storage time. However, after adjusting our models for difference in storage time between pre- and post-deployment samples (for both cases and controls), there were no changes in our adjusted means and *p*-values, so we opted for a more parsimonious model which did not include this variable.

Limitations of this study include lack of detailed information on deployment exposures for both the cases and controls. Deployment was used as a proxy for a PTE, and the exact timing of a PTE, if it occurred, is not known. There is also the issue that cases, by virtue of their PTSD diagnosis, were presumably exposed to a PTE, while we do not know the same for controls. In an attempt to investigate a possible bias from this disparity, we re-ran all our analyses utilizing a control group comprised only of those likely involved in combat during deployment (the group specified in Table 1 as “more likely involved in combat”). The methylation patterns we found did not differ from those using the complete control group (data not shown). We also tried to control for potential differences in deployment experience by ensuring that all cases and controls had not been previously deployed, that they were all active duty Army or Marines, and that they were deployed from 6 to 18 months, but there is still potential for significant variation among all our subjects with respect to intensity of combat during deployment. We do not have any data on previous exposure to a PTE or underlying personality traits, which have been hypothesized to influence vulnerability to PTSD (van Zuiden et al., 2011). Military personnel, prior to deployment, undergo a medical exam and are required to complete a pre-deployment health assessment survey (Department of Defense form 2795) to ensure medical readiness for deployment. This would have applied to all subjects in our study.

The timing of sample collection was not standardized, so there was heterogeneity in the length of time between deployment and serum draw. Although we tried to minimize this interval, the timing of each serum sample being added to the DoDSR depended on the timing of HIV testing for each military service member. We do not have data on other relevant exposures which may influence DNA methylation, such as dietary factors (folate, vitamin B_12_ intake) (Fenech, 2001; Piyathilake and Johanning, 2002) smoking (Toh et al., 2010), and alcohol consumption (Seitz and Stickel, 2007; Toh et al., 2010; Zhu et al., 2012).

Ascertainment of PTSD via query of ICD-9 coded medical encounter is not ideal. Although we attempted to restrict the definition of PTSD to a scenario which would minimize misclassification of disease, by requiring the ICD-9 code 309.81 be present in the first diagnostic position for two outpatient records spaced at a reasonable calendar time distance, this type of case ascertainment is still prone to misclassification. However, this is the PTSD case definition developed in September 2008 by the Department of Defense Interagency PTSD and TBI Standardization Committee, and it has been accepted by Military Health Affairs for surveillance (Armed Forces Health Surveillance Center, 2011). The DNA yields from the sera in this study were small, another potential limitation.

The limitations mentioned above are offset by the rare access to both pre- and post-deployment biologic samples in cases and controls. While most case-control studies would not be able to infer whether the observed methylation patterns were a consequence of PTSD or whether they indicated vulnerabilities that existed among the cases before the onset of PTSD, our study was able to address both possibilities. Another strength of the study is that we utilized pyrosequencing to provide a quantitative measure of DNA methylation. Pyrosequencing is a quantitative real-time sequencing technology. The light output or the Pyrosequencing signals (represent as Relative Light Unit or RLU) are directly proportional to the number of molecules being sequenced. Pyrosequencing QCpG software has a number of quality assessments to pass or to fail results for each sample being sequenced. These specifications give information including sample quality, non-specific PCR amplification, non-specific sequencing, and completion of bisulfite modification. Therefore, if a pyrosequencing run on a specific sample gave a passing result, it indicates that the DNA of that sample is of sufficient quality.

Chance could account for some of the statistically significant findings we report, given the number of comparisons and the small sample size, but this was an exploratory study and the results should be considered as preliminary. The DoDSR, which contains over 45 million longitudinally collected serum samples of U.S. military service members will provide a vast resource for carrying out future investigations in larger populations. There is growing evidence that the molecular mechanisms that regulate DNA methylation are involved in synaptic plasticity, learning, and memory (Sananbenesi and Fischer, 2009). DNA is inherently stable, compared with RNA, thus development of biomarkers based on DNA provide an attractive potential. Additionally, since modifications in DNA methylation can potentially be reversed – by de-methylating or methylating agents – understanding the role of DNA methylation in PTSD has the potential to fuel novel approaches to PTSD treatment.

## Conclusion

We evaluated DNA methylation from serum as a potential biomarker for PTSD. In this exploratory study of deployed personnel, comparing those with PTSD and those who did not develop PTSD, the latter had reduced %5-mC levels of H19 and IL18 after deployment and elevated levels of IL18 after deployment. Additionally, pre-deployment the people who later became cases had lower levels of IL18 %5-mC compared with controls. Whether these are markers of vulnerability or resilience is a matter of speculation, but because of the high incidence of PTSD, it is important to study these patterns further.

## Conflict of Interest Statement

The authors declare that the research was conducted in the absence of any commercial or financial relationships that could be construed as a potential conflict of interest.

## Supplementary Material

The Supplementary Material for this article can be found online at http://www.frontiersin.org/Molecular_Psychiatry/10.3389/fpsyt.2013.00056/abstract

Supplementary Table S1 **Generalized linear models for investigating the adjusted means^∗^ and differences between controls pre- and post-deployment and cases pre- and post-deployment**.

Supplementary Table S2 **Generalized linear models investigating the adjusted means^∗^ and differences between cases and controls, pre-deployment and post-deployment**.
