# Supplementary material for: PTSD and DNA Methylation in Select Immune Function Gene Promoter Regions: A Repeated Measures Case-Control Study of U.S. Military Service Members
Source: Front Psychiatry. 2013 Jun 24;4:56. doi: 10.3389/fpsyt.2013.00056 (PMC3690381; doi:10.3389/fpsyt.2013.00056)
Supplement: Supplementary Table S1 — Generalized linear models for investigating the adjusted means∗ and differences between controls pre- and post-deployment and cases pre- and post-deployment. [file 48787_Rusiecki_DataSheet1.DOCX]

| **Appendix A. Generalized Linear Models for investigating the adjusted means* and differences between controls pre- and post-deployment and cases pre- and post-deployment** | | | | | | | | |
| --- | --- | --- | --- | --- | --- | --- | --- | --- |
|  |  |  |  |  |  |  |  |  |
|  |  | **Controls** | | |  | **Cases** | | |
| **Gene/locus** |  | N | mean* | p-value |  | N | mean* | p-value |
| **IGF-2 ^a^** mean of positions 1-6 | Pre | 62 | 39.69 | 0.62 |  | 52 | 43.45 | 0.96 |
| (TSS-479, 476, 460, 361, 341, 322) | Post | 62 | 40.91 |  |  | 52 | 43.30 |  |
|  |  |  |  |  |  |  |  |  |
| **H-19** mean of positions 1-4 | Pre | 45 | 56.79 | 0.01 |  | 37 | 56.08 | 0.93 |
|  | Post | 45 | 54.25 |  |  | 37 | 55.95 |  |
|  |  |  |  |  |  |  |  |  |
| H-19 Pos1 (TSS-1964) | Pre | 45 | 53.30 | 0.03 |  | 37 | 52.57 | 0.92 |
|  | Post | 45 | 50.97 |  |  | 37 | 52.76 |  |
|  |  |  |  |  |  |  |  |  |
| H-19 Pos2 (TSS-1946) | Pre | 45 | 58.44 | 0.14 |  | 37 | 56.98 | 0.75 |
|  | Post | 45 | 56.75 |  |  | 37 | 57.62 |  |
|  |  |  |  |  |  |  |  |  |
| H-19 Pos3 (TSS-1927) | Pre | 45 | 55.31 | 0.01 |  | 37 | 52.86 | 0.72 |
|  | Post | 45 | 52.48 |  |  | 37 | 53.44 |  |
|  |  |  |  |  |  |  |  |  |
| H-19 Pos4 (TSS-1919) | Pre | 45 | 60.12 | 0.05 |  | 37 | 61.91 | 0.25 |
|  | Post | 45 | 56.81 |  |  | 37 | 59.97 |  |
|  |  |  |  |  |  |  |  |  |
|  |  |  |  |  |  |  |  |  |
| **IL-8 ^c^** mean of positions 1-3 | Pre | 44 | 3.00 | 0.18 |  | 36 | 2.54 | 0.99 |
| (TSS-116, -106, -31) | Post | 44 | 2.57 |  |  | 36 | 2.54 |  |
|  |  |  |  |  |  |  |  |  |
|  |  |  |  |  |  |  |  |  |
| **IL-16  ^d^** mean of positions 1-4 | Pre | 38 | 28.47 | 0.90 |  | 31 | 24.48 | 0.56 |
| (TSS-159, -139, -93, -79) | Post | 38 | 28.05 |  |  | 31 | 22.46 |  |
|  |  |  |  |  |  |  |  |  |
| **IL-18** mean of positions 1-5 | Pre | 45 | 15.34 | 0.11 |  | 37 | 11.93 | 0.15 |
|  | Post | 45 | 12.73 |  |  | 37 | 14.59 |  |
|  |  |  |  |  |  |  |  |  |
| IL-18 pos1 (TSS-158) | Pre | 44 | 41.50 | 0.30 |  | 37 | 39.54 | 0.93 |
|  | Post | 44 | 39.14 |  |  | 37 | 39.75 |  |
|  |  |  |  |  |  |  |  |  |
| IL-18 pos2 (TSS-108) | Pre | 44 | 8.70 | 0.27 |  | 37 | 4.77 | 0.13 |
|  | Post | 44 | 6.61 |  |  | 37 | 8.62 |  |
|  |  |  |  |  |  |  |  |  |
| IL-18 pos3 (TSS-86) | Pre | 44 | 7.28 | 0.79 |  | 37 | 5.34 | 0.25 |
|  | Post | 44 | 6.77 |  |  | 37 | 8.20 |  |
|  |  |  |  |  |  |  |  |  |
| IL-18 pos4 (TSS-49) | Pre | 43 | 9.89 | 0.18 |  | 37 | 6.70 | 0.06 |
|  | Post | 43 | 7.59 |  |  | 37 | 10.19 |  |
|  |  |  |  |  |  |  |  |  |
| IL-18 pos5 (TSS-33) | Pre | 42 | 7.45 | 0.04 |  | 37 | 3.28 | 0.16 |
|  | Post | 42 | 4.15 |  |  | 37 | 6.18 |  |
| * adjusted for age (younger, older), gender, and race (black, white) | | | | | |  |  |  |
| ^a^ results for individual positions for IGF-2 are all non-statistically significant, so results for only the mean of the positions are presented | | | | | | | | |
| ^b^ results for individual positions for EDG-1 are all non-statistically significant, so results for only the mean of the positions are presented | | | | | | | | |
| ^c^ results for individual positions for IL-8 are all non-statistically significant, so results for only the mean of the positions are presented | | | | | | | | |
| ^d^ results for individual positions for IL-16 are all non-statistically significant, so results for only the mean of the positions are presented | | | | | | | | |

| **Appendix B. Generalized Linear Models investigating the adjusted means* and differences between cases and controls, pre-deployment and post-deployment** | | | | | | | | |
| --- | --- | --- | --- | --- | --- | --- | --- | --- |
|  |  |  |  |  |  |  |  |  |
|  |  | **Pre-deployment** | | |  | **Post-deployment** | | |
| **Gene/locus** |  | N | mean* | p-value |  | N | mean* | p-value |
| **IGF-2** mean of positions 1-6 | case | 62 | 45.83 | 0.12 |  | 63 | 42.96 | 0.08 |
|  | control | 71 | 42.10 |  |  | 65 | 38.29 |  |
|  |  |  |  |  |  |  |  |  |
| IGF-2 pos1 (TSS-479) | case | 62 | 43.76 | 0.08 |  | 63 | 39.60 | 0.16 |
|  | control | 71 | 37.95 |  |  | 65 | 34.80 |  |
|  |  |  |  |  |  |  |  |  |
| IGF-2 pos2 (TSS-476) | case | 62 | 65.32 | 0.01 |  | 63 | 59.21 | 0.22 |
|  | control | 71 | 56.48 |  |  | 65 | 54.34 |  |
|  |  |  |  |  |  |  |  |  |
| IGF-2 pos3 (TSS-460) | case | 62 | 47.46 | 0.22 |  | 63 | 50.31 | 0.07 |
|  | control | 71 | 42.83 |  |  | 65 | 42.73 |  |
|  |  |  |  |  |  |  |  |  |
| IGF-2 pos4 (TSS-361) | case | 61 | 49.79 | 0.38 |  | 62 | 42.25 | 0.14 |
|  | control | 69 | 46.67 |  |  | 65 | 37.04 |  |
|  |  |  |  |  |  |  |  |  |
| IGF-2 pos5 (TSS-341) | case | 61 | 37.31 | 0.57 |  | 62 | 35.45 | 0.49 |
|  | control | 69 | 39.04 |  |  | 65 | 33.45 |  |
|  |  |  |  |  |  |  |  |  |
| IGF-2 pos6 (TSS-322) | case | 61 | 30.59 | 0.53 |  | 62 | 30.13 | 0.31 |
|  | control | 69 | 29.01 |  |  | 65 | 27.46 |  |
|  |  |  |  |  |  |  |  |  |
| **H-19 ^a^** mean of positions 1-4 | case | 53 | 55.93 | 0.68 |  | 50 | 56.16 | 0.24 |
| (TSS-1964, -1946, -1927, -1919) | control | 60 | 56.42 |  |  | 51 | 54.78 |  |
|  |  |  |  |  |  |  |  |  |
| **IL-8** mean of positions 1-3 | case | 52 | 2.72 | 0.07 |  | 50 | 2.64 | 0.57 |
|  | control | 59 | 3.19 |  |  | 51 | 2.48 |  |
|  |  |  |  |  |  |  |  |  |
| IL-8 pos1 (TSS-116) | case | 52 | 2.91 | 0.23 |  | 50 | 2.47 | 0.64 |
|  | control | 59 | 3.46 |  |  | 51 | 2.28 |  |
|  |  |  |  |  |  |  |  |  |
| IL-8 pos2 (TSS-106) | case | 52 | 2.77 | 0.01 |  | 50 | 2.87 | 0.79 |
|  | control | 59 | 3.62 |  |  | 51 | 2.76 |  |
|  |  |  |  |  |  |  |  |  |
| IL-8 pos3 (TSS-31) | case | 52 | 2.48 | 0.90 |  | 50 | 2.58 | 0.22 |
|  | control | 59 | 2.49 |  |  | 51 | 2.41 |  |
|  |  |  |  |  |  |  |  |  |
| **IL-16  ^c^** mean of positions 1-4 | case | 48 | 27.30 | 0.84 |  | 47 | 23.05 | 0.99 |
| (TSS-159, -139, -93, -79) | control | 56 | 27.86 |  |  | 52 | 23.06 |  |
|  |  |  |  |  |  |  |  |  |
| **IL-18** mean of positions 1-5 | case | 53 | 11.91 | <0.01 |  | 50 | 14.02 | 0.46 |
|  | control | 59 | 15.78 |  |  | 50 | 12.65 |  |
|  |  |  |  |  |  |  |  |  |
| IL-18 pos1 (TSS-158) | case | 53 | 38.98 | 0.08 |  | 50 | 40.68 | 0.32 |
|  | control | 59 | 42.61 |  |  | 49 | 38.29 |  |
|  |  |  |  |  |  |  |  |  |
| IL-18 pos2 (TSS-108) | case | 53 | 5.24 | 0.01 |  | 50 | 7.68 | 0.44 |
|  | control | 58 | 9.07 |  |  | 49 | 5.89 |  |
|  |  |  |  |  |  |  |  |  |
| IL-18 pos3 (TSS-86) | case | 53 | 5.54 | 0.06 |  | 50 | 7.48 | 0.59 |
|  | control | 58 | 7.80 |  |  | 49 | 6.19 |  |
|  |  |  |  |  |  |  |  |  |
| IL-18 pos4 (TSS-49) | case | 53 | 6.47 | <0.01 |  | 50 | 9.60 | 0.15 |
|  | control | 58 | 10.17 |  |  | 48 | 6.91 |  |
|  |  |  |  |  |  |  |  |  |
| IL-18 pos5 (TSS-33) | case | 53 | 3.79 | <0.01 |  | 50 | 5.91 | 0.19 |
|  | control | 58 | 7.64 |  |  | 47 | 3.59 |  |
| * adjusted for age (younger, older), gender, and race (black, white) | | | | | |  |  |  |
| ^a^ results for individual positions for IGF-2 are all non-statistically significant and are not presented in the table | | | | | | | | |
| ^b^ results for individual positions for IL-8Rα are all non-statistically significant and are not presented in the table | | | | | | | | |
| ^c^ results for individual positions for IL-16 are all non-statistically significant and are not presented in the table | | | | | | | | |
